# Tissue Trace Elements and Lipid Peroxidation in Breeding Female Bank Voles *Myodes glareolus*

**DOI:** 10.1007/s12011-018-1353-7

**Published:** 2018-04-27

**Authors:** Elżbieta Bonda-Ostaszewska, Tadeusz Włostowski, Barbara Łaszkiewicz-Tiszczenko

**Affiliations:** Institute of Biology, University of Bialystok, Ciołkowskiego 1J, 15-245 Białystok, Poland

**Keywords:** Bank vole, Lipid peroxidation, Iron, Copper, Zinc, Reproduction

## Abstract

Recent studies have demonstrated that reproduction reduces oxidative damage in various tissues of small mammal females. The present work was designed to determine whether the reduction of oxidative stress in reproductive bank vole females was associated with changes in tissue trace elements (iron, copper, zinc) that play an essential role in the production of reactive oxygen species. Lipid peroxidation (a marker of oxidative stress) and iron concentration in liver, kidneys, and skeletal muscles of reproducing bank vole females that weaned one litter were significantly lower than in non-reproducing females; linear regression analysis confirmed a positive relation between the tissue iron and lipid peroxidation. The concentrations of copper were significantly lower only in skeletal muscles of reproductive females and correlated positively with lipid peroxidation. No changes in tissue zinc were found in breeding females when compared with non-breeding animals. These data indicate that decreases in tissue iron and copper concentrations may be responsible for the reduction of oxidative stress in reproductive bank vole females.

## Introduction

Reproduction is the most energetically costly period of the female’s life [1]. In small mammals, energy intake typically increases by 66–180%, and metabolic rate (oxygen consumption) rises twofold in lactating females when compared with non-reproductives [2–4]. This has led to the suggestion that the production of reactive oxygen species (ROS), created primarily by mitochondria as a byproduct of ATP synthesis, should also be elevated, resulting in oxidative damage to lipids, proteins, and DNA [5]. Contrary to this suggestion, recent studies have demonstrated that oxidative damage in various tissues of breeding female house mice *Mus musculus* [6], bank voles *Myodes glareolus* [7, 8], and Damaraland mole-rats *Fukomys damarensis* [9] is lower when compared with non-reproductive controls. However, the exact mechanism underlying the reduction of oxidative stress in breeding females remains to be elucidated.

Oxidative stress can be defined as an excessive bioavailability of ROS, which is the net result of an imbalance between production and destruction of ROS, the latter being influenced by antioxidant defenses [10]. Importantly, the reduction of oxidative stress in various tissues of the breeding females mentioned above has been found not to correlate clearly with antioxidant defenses such as superoxide dismutase, catalase, or glutathione [6, 8, 9]. Thus, it may be concluded that a low intensity of ROS production rather than destruction is responsible for the phenomenon. Iron (Fe) and copper (Cu) play an important role in the production of ROS, which mediate catalysis of hydroxyl radicals (HO^•^) from hydrogen peroxide (H_2_O_2_) [11, 12]. It has been well documented that any reductions in the concentrations of labile iron ions decrease oxidative stress in cultured cells [13]. Likewise, dietary cadmium has been shown to decrease liver and kidney lipid peroxidation (a sensitive marker of oxidative stress) below control values in bank voles and Swiss mice, through lowering the tissue iron concentrations [14, 15]. Notably, iron depletion in various tissues of reproductive rat females has also been found, but without measuring any markers of oxidative damage [16]. Based on these observations, one may hypothesize that the reduction of oxidative stress in reproducing females is associated with decreased tissue iron concentration brought about by reproduction.

To test this hypothesis, we used the bank vole females because the reproduction appeared to reduce tissue oxidative damage in this species [7, 8]. In the present work, lipid peroxidation in the liver, kidneys, and skeletal muscles of reproducing and non-reproducing bank vole females was determined; at the same time, iron, copper—another prooxidant element [12]—and zinc known as antioxidant [17] were examined, to establish a relationship between these elements and lipid peroxidation.

## Materials and Methods

### Animals and Experimental Design

Immature females (with closed vagina) and males (with undeveloped testes) of the bank vole (weight 12.5–14.0 g) were captured in September 2015 in live traps in the Knyszyn Old Forest near Białystok (northeastern Poland). The animals were then housed individually for 5 months in standard plastic mouse cages (27 × 21 × 14 cm) lined with sawdust as absorptive material and hay in the nest compartment at a constant temperature (20 ± 1 °C) and long photoperiod (16 h light/8 h dark). They received ad libitum tap water and whole wheat grains; in addition, an identical quantity of apple was offered to all animals (3 g/vole/week), who ate it completely [18]. Atomic absorption spectrophotometry (AAS) analysis of the grain revealed that actual levels of iron, copper, and zinc were 80–100, 4–6, and 22–26 μg/g of dry weight, respectively.

In February 2016, 20 mature females (weighing 16.5–18.0 g) were paired with males, which were removed from the cage before parturition (a reproductive group). Another group of females were not paired with males and kept until the end of lactation of the reproductives (a non-reproductive group). Breeding pairs were kept in larger cages (43 × 27 × 16 cm) with plastic houses for nesting, in the same room as non-breading animals. Twenty one days after parturition (the end of lactation), ten females that weaned three to five pups and ten virgins were weighed and euthanized by cervical dislocation and subsequent decapitation. During this section, the voles were exsanguinated by heart puncture, and the liver, kidneys, and gastrocnemius muscle were removed, cut into small pieces, rinsed in cold saline, blotted dry on absorbent paper, and kept at − 80 °C until analysis. All the experimental procedures were approved by the local ethical committee in Medical University of Białystok (permission number: 70/2015).

### Lipid Peroxidation Assay

Lipid peroxidation was assessed by measuring malondialdehyde (MDA) formation, using the thiobarbituric acid (TBA) assay [19]. After thawing, a portion of the liver, kidneys, and skeletal muscles (about 200 mg) was transferred to 1.0 ml chilled 1.15% KCl and homogenized with Teflon pestle in a glass homogenizer. 0.1 ml of the tissue homogenate, 0.2 ml of 8.1% sodium dodecyl sulfate, 1.5 ml of 20% acetic acid, 1.5 ml of 0.8% TBA, and 0.6 ml of distilled water were added and vortexed. The reaction mixture was placed in a water bath at 95 °C for 1 h. After cooling, 1.0 ml of distilled water and 5.0 ml of butanol/pyridine mixture (15:1 *v*/*v*) were added and vortexed. After centrifugation, the absorbance of the organic phase was determined at 532 nm. Tetraethoxypropane was used to prepare a calibration curve. The results were expressed as TBA-reactive substances (TBARS) (nmol/g wet weight).

### Trace Elements Determination

Metal determinations were performed as described recently in Włostowski et al. [20]. The homogenate (0.6 ml) was digested at 200 °C for 40 min in a mixture of redistilled nitric acid (70%) (Sigma-Aldrich) (2.5 ml), 30% H_2_O_2_ (Sigma-Aldrich) (0.25 ml), and deionized water (1.65 ml), using a Mars 6 microwave oven (CEM Corporation, Matthews, NC, USA). A portion of the obtained solution (250 μl) was evaporated to dryness in a quartz crucible at 70 °C and the residue was redissolved in an appropriate amount of deionized water. Metal analyses of these solutions were carried out by electrothermal AAS using a Thermo iCE 3400 instrument with Zeeman correction (Thermo Electron Manufacturing Ltd., Cambridge, UK). Quality assurance procedures included the analysis of reagent blanks and standard reference material (Bovine liver 1577c—the National Institute of Standards and Technology, Gaithersburg, MD). The recoveries of iron, copper, and zinc were 95–101, 89–95, and 90–95%, respectively.

### Statistical Analysis

The data were expressed as mean ± SD. In order to meet parametric assumptions, all data were log transformed. Two-way analysis of variance (ANOVA) was performed to examine effects of reproductive state, tissue type, and their interaction on TBARS and trace element levels. The Student *t* test was then applied to determine the significance of difference between reproductive and non-reproductive females. The relationship between the concentration of TBARS and the levels of iron, copper, and zinc was tested by using linear regression analysis. All the statistical analyses were performed using IBM SPSS Statistics 24.0.

## Results

Body mass, recorded at the end of lactation, was significantly higher in females that weaned one litter (three to five pups) (22.3 ± 4.1 g, *n* = 10) than body mass measured at the same time in non-reproducing females (17.5 ± 2.5 g, *n* = 10) (*P* = 0.0091). Absolute mass of liver and kidneys also was significantly higher in reproductive than in non-reproductive females (Table 1).

**Table 1:** Organ mass, lipid peroxidation (TBARS), and trace elements levels in the liver, kidneys, and skeletal muscles of reproductive and non-reproductive female bank voles

	Reproductive	Non-reproductive	Student’s *t* test (*P* value)
Liver mass (g)	1.68 ± 0.28	0.81 ± 0.18	< *0.0001*
Kidney mass (g)	0.26 ± 0.03	0.20 ± 0.03	*0.0014*
TBARS (nmol/g)			
Liver	46.9 ± 4.0	92.9 ± 19.5	< *0.0001*
Kidneys	76.9 ± 4.7	148.3 ± 49.3	*0.0001*
Muscle	88.7 ± 39.8	390.1 ± 183.7	*0.0002*
Iron (μg/g)			
Liver	121.2 ± 15.6	163.9 ± 17.9	< *0.0001*
Kidneys	117.7 ± 9.1	213.0 ± 56.8	< *0.0001*
Muscle	52.2 ± 7.3	64.5 ± 6.1	*0.0001*
Copper (μg/g)			
Liver	2.24 ± 0.54	3.19 ± 1.41	0.0598
Kidneys	3.35 ± 0.63	3.08 ± 0.60	0.5717
Muscle	1.40 ± 0.22	2.76 ± 0.72	< *0.0001*
Zinc (μg/g)			
Liver	19.1 ± 2.2	23.1 ± 4.7	0.0793
Kidneys	20.6 ± 3.2	20.6 ± 2.7	0.9778
Muscle	9.8 ± 0.9	11.0 ± 1.8	0.0826

Values represent mean ± SD for *n* = 10. *P* values of statistically significant effects are shown in italics. The results of two-way ANOVA are presented in Table 2

Lipid peroxidation (TBARS) and iron and copper concentrations were significantly affected by reproductive state, tissue type, and their interaction (Tables 1 and 2). The levels of TBARS in liver, kidneys, and skeletal muscles were 49, 48, and 77%, respectively, lower in breeding than in non-breeding females. Likewise, the concentrations of iron in liver, kidneys, and skeletal muscles were significantly lower (26, 45, and 19%, respectively) in reproductive than in non-reproductive females. The concentrations of copper in liver and kidneys were similar in reproducing and non-reproducing females, but its level in skeletal muscles was significantly lower (49%) in breeding than in non-breeding bank voles. The levels of zinc in all organs did not differ significantly between reproductive and non-reproductive females (Table 1).

**Table 2:** Variation in lipid peroxidation (TBARS) and trace elements in relation to reproductive state and tissue (two-way ANOVA)

	Source of variability	*F*	*P*
TBARS	Reproductive state	40.719	< *0.0001*
	Tissue	14.223	< *0.0001*
	Reproductive state × tissue	3.251	*0.0487*
Iron	Reproductive state	58.67	< *0.0001*
	Tissue	179.33	< *0.0001*
	Reproductive state × tissue	4.94	*0.0119*
Copper	Reproductive state	12.1376	*0.0012*
	Tissue	12.1799	*0.0001*
	Reproductive state × tissue	6.7113	*0.0030*
Zinc	Reproductive state	2.03	0.1183
	Tissue	112.45	< *0.0001*
	Reproductive state × tissue	1.49	0.2372

*P* values of statistically significant effects are shown in italics

**Fig. 1:**
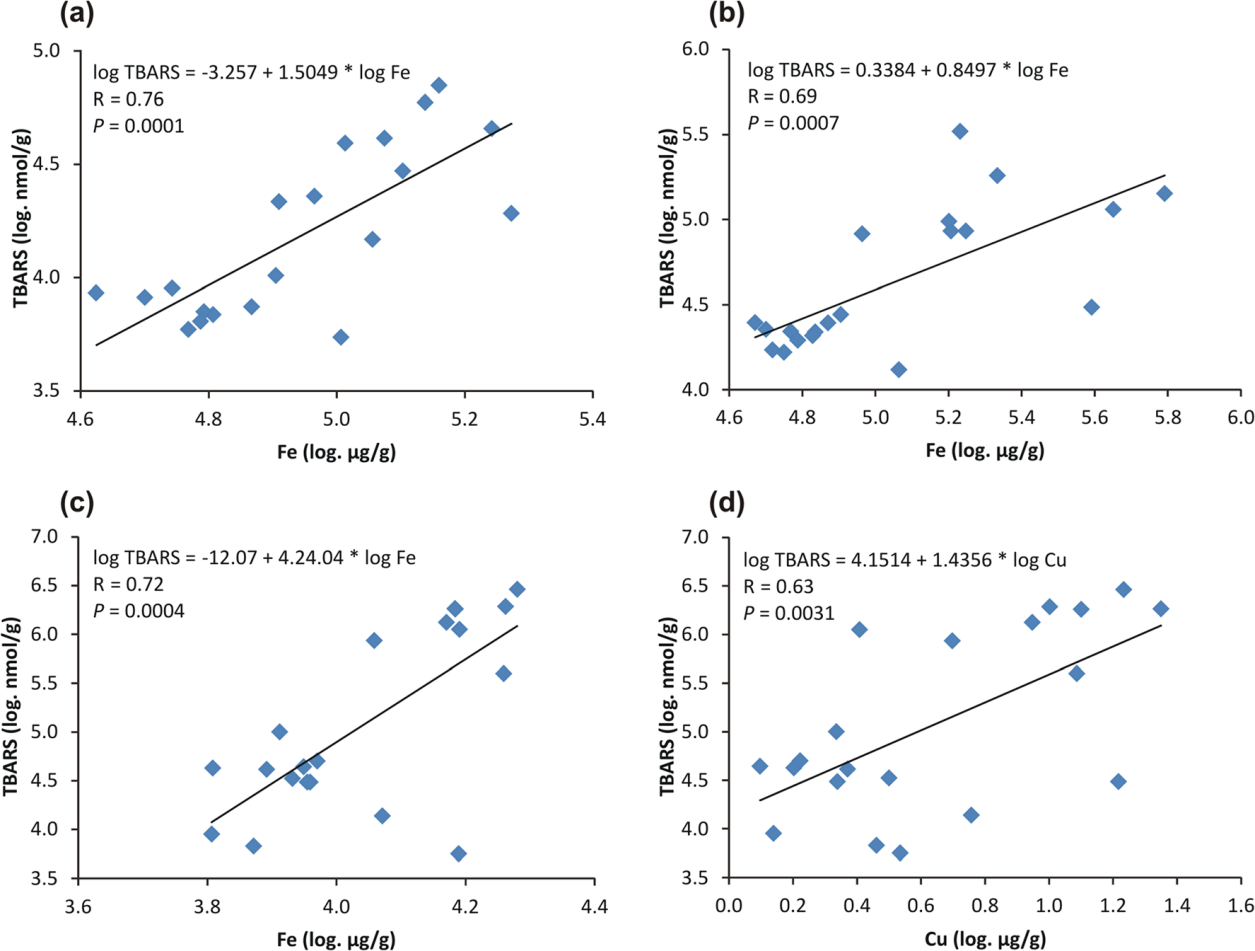
The relationship of tissue iron (Fe) and copper (Cu) to lipid peroxidation in **a** liver, **b** kidneys, and **c**, **d** skeletal muscle of female bank voles

Linear regression analysis revealed a positive correlation between the liver, kidneys, and muscle iron concentration and lipid peroxidation (TBARS) (Fig. 1a–c). A positive relationship was also found between the muscle copper concentration and lipid peroxidation (Fig. 1d), but no correlation was observed between the tissue zinc and TBARS level (*P* = 0.08–0.98). Notably, the same analysis showed an inverse correlation between the liver and kidney absolute mass and lipid peroxidation: *R* = − 0.82, *P* < 0.0001 and *R* = − 0.62, *P* = 0.0099, respectively.

## Discussion

The results of the present study confirmed previous observations that reproduction (pregnancy and lactation) reduces oxidative damage in various tissues of small mammal females and thus questioned the hypothesis that oxidative stress is a mechanism underlying the cost of reproduction, e.g., decreased survival [6–9]. The notion is supported by the fact that reproductive Damaraland mole-rat females that exhibit lower tissue oxidative damage live longer than non-reproductive females [9]. Whether or not reproduction (and lower oxidative stress) also increases the longevity in other mammals remains to be determined. Also, the exact mechanism underlying the reduction of oxidative stress in reproducing females is not clear. It has been proposed that ROS are neutralized by antioxidant defenses induced in reproductive females [7], but other studies demonstrated that activities (levels) of superoxide dismutase, catalase, and glutathione in most tissues appear not to correlate clearly with oxidative damage [6, 8, 9]. Thus, other mechanisms may also be responsible for the reduction of oxidative stress in reproducing females.

The present study also showed that during reproduction, the concentration of iron decreases in the liver, kidneys, and skeletal muscles, the level of copper declines only in skeletal muscles, and zinc content undergoes no significant changes in the bank vole females (Table 1). The depletion of tissue iron concentrations in reproducing females appears to be due to a very high demand of fetal and postnatal development for this element [21], which cannot be fully compensated by absorptive processes in the gastrointestinal tract despite hyperphagia and an adaptive increase in the efficiency of iron absorption induced by the decreasing tissue iron status [16]. In contrast, the demand for zinc and copper during reproduction is not so marked as for iron and seems to be relatively well covered by the increase in food consumption as well as by an increase in the ability to absorb zinc and copper in the intestine [16]. Still, a mechanism underlying a substantial decrease of copper concentration only in skeletal muscles of reproductive bank vole females is not known and remains to be clarified.

The present work revealed further that the reduction of lipid peroxidation (a marker of oxidative stress) in the liver, kidneys, and skeletal muscles of reproductive bank vole females was accompanied by a decrease in the tissue iron concentration; linear regression analysis confirmed a positive relation between the tissue iron and lipid peroxidation (Fig. 1). Since iron is known to catalyze the production of hydroxyl radicals in the Fenton reaction [11], it is possible that the reduction of oxidative stress in these females may be associated with lowering the tissue iron due to reproduction. The idea is in good agreement with other studies showing a close correlation between tissue iron and lipid peroxidation [14, 15, 22, 23]. It is worth noting that iron depletion is a common phenomenon also in pregnant women [11, 21]. Thus, one may assume that lowering the tissue iron content in the women should result in the reduction of oxidative stress. However, whether or not it is the case in humans remains to be determined.

It is well known that copper and zinc also play an essential role in oxidative stress; copper has been shown to enhance oxidative damage [24], while zinc can protect biomolecules from oxidation by inhibiting the production of ROS [17]. The present study showed that reproduction did not change tissue zinc levels, suggesting that this element was probably not responsible for the reduction of lipid peroxidation. However, our results do not exclude the possibility that in skeletal muscles of reproducing bank vole females, a decline in copper could be involved in the inhibition of oxidative damage. Notably, a concurrent decrease of copper and iron levels in skeletal muscles might well account for the highest reduction of lipid peroxidation observed in this tissue of reproducing females.

Although tissue iron and copper depletion may be involved, at least to some degree, in the reduction of lipid peroxidation in liver, kidneys, and skeletal muscles of reproductive bank vole females, the results of the present study suggest that changes in the organ mass may also contribute significantly to this process. Based on the correlation coefficients (the “Results” section), it may be concluded that the liver and kidney mass appears to be a significant factor affecting oxidative damage; the higher organ mass in reproductive females results in lower oxidative stress as compared to non-reproductive females. Unfortunately, this relationship cannot be easily explained at present; however, assuming that an increase in the organ mass observed in reproductive bank vole females [7, 8, present study] is associated with changes in cell size rather than their numbers [25], then the cells of higher size in these females should consume less oxygen and so produce less ROS (due to favorable surface area-to-volume ratio) as compared to non-reproductive females. However, this possible mechanism and an involvement of iron and copper in oxidative stress are not mutually exclusive and need to be studied in detail.

In conclusion, the present data indicate that the reduction of oxidative stress in liver, kidneys, and skeletal muscles of reproducing bank vole females may be associated with lowering tissue iron and copper concentrations, probably brought about by reproductive processes.
